# Application of Defined Approaches to Assess Skin Sensitization Potency of Isothiazolinone Compounds

**DOI:** 10.1089/aivt.2022.0014

**Published:** 2022-12-16

**Authors:** Judy Strickland, David G. Allen, Dori Germolec, Nicole Kleinstreuer, Victor J. Johnson, Travis Gulledge, Jim Truax, Anna Lowit, Timothy Dole, Timothy McMahon, Melissa Panger, Judy Facey, Stephen Savage

**Affiliations:** ^1^ Inotiv, Inc., Morrisville, North Carolina, USA.; ^2^ Division of Translational Toxicology, National Institute of Environmental Health Sciences, Research Triangle Park, North Carolina, USA.; ^3^ National Toxicology Program Interagency Center for the Evaluation of Alternative Methods, National Institute of Environmental Health Sciences, Research Triangle Park, North Carolina, USA.; ^4^ Burleson Research Technologies, Inc., Morrisville, North Carolina, USA.; ^5^ United States Environmental Protection Agency, Office of Pollution Prevention and Toxics, Washington, District of Columbia, USA.; ^6^ United States Environmental Protection Agency, Office of Pesticide Programs, Washington, District of Columbia, USA.

**Keywords:** artificial neural network, LLNA, nonanimal methodology, point of departure, risk assessment, skin sensitization

## Abstract

**Introduction:**

Isothiazolinones (ITs) are widely used as antimicrobial preservatives in cosmetics and as additives for preservation of consumer and industrial products to control bacteria, fungi, and algae. Although they are effective biocides, they have the potential to produce skin irritation and sensitization, which poses a human health hazard. In this project, we evaluated nonanimal defined approaches (DAs) for skin sensitization that can provide point-of-departure estimates for use in quantitative risk assessment for ITs.

**Materials and Methods:**

The skin sensitization potential of six ITs was evaluated using three internationally harmonized nonanimal test methods: the direct peptide reactivity assay, KeratinoSens™, and the human cell line activation test. Results from these test methods were then applied to two versions of the Shiseido Artificial Neural Network DA.

**Results:**

Sensitization hazard or potency predictions were compared with those of the in vivo murine local lymph node assay (LLNA). The nonanimal methods produced skin sensitization hazard and potency classifications concordant with those of the LLNA. EC3 values (the estimated concentration needed to produce a stimulation index of three, the threshold positive response) generated by the DAs had less variability than LLNA EC3 values, and confidence limits from the DAs overlapped those of the LLNA EC3 for most substances.

**Conclusion:**

The application of *in silico* models to *in chemico* and in vitro skin sensitization data is a promising data integration procedure for DAs to support hazard and potency classification and quantitative risk assessment.

## Introduction

Nonanimal test methods for regulatory assessment of skin sensitization potential include *in chemico* and in vitro test methods based on the adverse outcome pathway (AOP) for skin sensitization.^1,2^ These tests cannot be used alone to determine skin sensitization potential but are used in combination with one another in defined approaches (DAs). DAs consist of a fixed data interpretation procedure applied to a specific set of information sources to satisfy a regulatory need.^3,4^ An important limitation of the DAs currently accepted for regulatory use is that they provide information only on skin sensitization hazard and potency classification.^4^ Although there are methods for developing quantitative points of departure for skin sensitization risk assessment that use in vivo data, there are no regulatory test guidelines for nonanimal methods to support quantitative risk assessment.

Quantitative methods to estimate risk of skin sensitization were first examined by the U.S. Environmental Protection Agency's (U.S. EPA) Office of Pesticide Programs in 2004 using the available in vivo methods at that time, primarily the murine local lymph node assay (LLNA), and human studies approved for use by the agency's Human Studies Review Board.^5^ Such methods can be used to identify the dermal dose of a substance that is not expected to either induce skin sensitization in animals or humans not previously exposed (induction threshold) or elicit skin sensitization in animals or humans previously sensitized (elicitation threshold) to the chemical of interest.

Induction threshold doses can be determined from the LLNA or from human predictive patch tests conducted on normal human volunteers, typically the human repeat insult patch test. Elicitation thresholds can also be determined from human patch test studies, and from animal studies if appropriately designed. Since the initial examination of quantitative methods to assess dermal sensitization, advancements in both available technologies and acceptance of alternatives to animal testing have progressed such that it is now appropriate to examine use of *in chemico*, in vitro, and *in silico* test methods for this purpose.

Our objective for this project was to evaluate DAs for skin sensitization that can provide point-of-departure estimates for use in quantitative risk assessment. We tested six isothiazolinone (IT) compounds identified as a case study by the U.S. EPA and the IT Task Force of the American Chemistry Council. These compounds are widely used as preservatives in household cleaning products, latex paints, pressure-treated wood, metal working fluids, textiles, and plastics. There is considerable evidence that ITs produce allergic contact dermatitis^6,7^ and there is a need to quantify the potency of this chemical class for inducing skin sensitization.

A quantitative risk assessment is necessary to establish safe exposure levels that avoid inducing skin sensitization for products that do not bear warning language on labels. We developed a nonanimal quantitative assessment of skin sensitization by applying two artificial neural network (ANN) models^8^ to data generated by in vitro and *in chemico* skin sensitization assays. These models estimate murine LLNA EC3 values (the estimated concentrations needed to produce a stimulation index of three, the threshold positive response), which can be used as points of departure. We then compared the nonanimal estimates of EC3 values with those from historical animal data.

## Materials and Methods

### IT compounds tested

Table 1 provides the identity, structure, source, and purity information for the IT compounds tested.

**Table 1.** Isothiazolinone Compounds Tested

Common name			Structure
	Chemical name	CASRN	
DCOIT	4,5-Dichloro-2-octyl-3(2H)-isothiazolone	64359-81-5	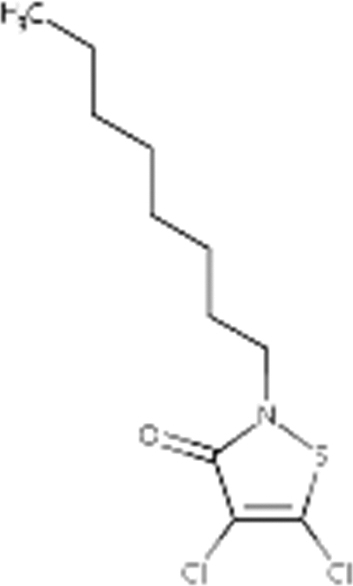
	*Product name (source)*	*% Active ingredient*	
	KATHON™ 287T Industrial Microbicide (Dow Chemical Company)	99.3	
	*Chemical name*	*CASRN*	
CMIT/MIT	Mixture of 5-chloro-2-methyl-4-isothiazolin-3-one and 2-methyl-4-isothiazolin-3-one	55965-84-9	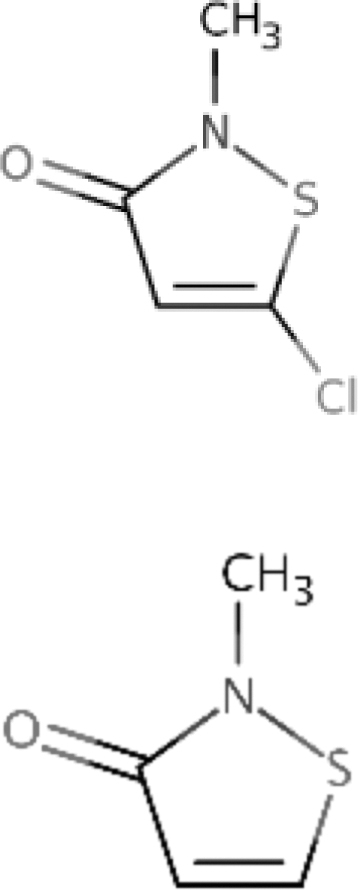
	*Product name (source)*	*% Active ingredient*	
	MERGAL MITZ (Troy Chemical Corporation)	14.2	
	*Chemical name*	*CASRN*	
OIT	2-*n*-Octyl-4-isothiazolin-3-one	26530-20-1	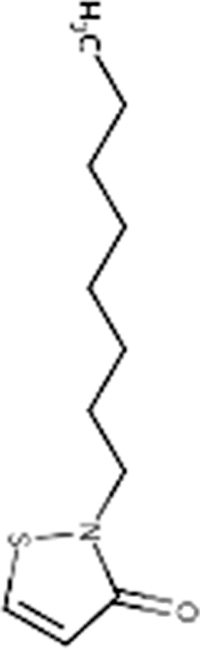
	*Product name (source)*	*% Active ingredient*	
	ACTICIDE OIT (Thor Specialties)	98.13	
	*Chemical name*	*CASRN*	
MIT	2-Methyl-4-isothiazolin-3-one	2682-20-4	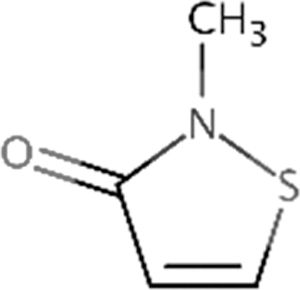
	*Product name (source)*	*% Active ingredient*	
	KORDEK™ 573F Industrial Microbiocide (Dow Chemical Company)	50.8	
BIT	1,2-Benzisothiazolin-3-one	2634-33-5	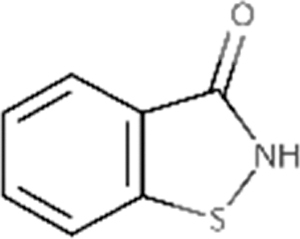
	*Product name (source)*	*% Active ingredient*	
	MERGAL BIT Technical (Troy Chemical Corporation)	85.2	
	*Chemical name*	*CASRN*	
BBIT	1,2-Benzisothiazolin-3-one, 2-butyl	4299-07-4	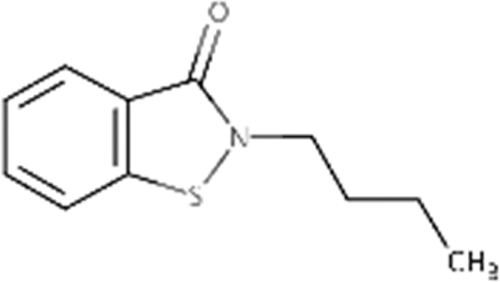
	*Product name (source)*	*% Active ingredient*	
	VANQUISH 100 (Lonza)	98.4	

### Nonanimal test methods for skin sensitization

The skin sensitization potential of the six ITs were evaluated using the *in chemico* direct peptide reactivity assay (DPRA), the in vitro KeratinoSens™ assay, and the in vitro human cell line activation test (h-CLAT). Testing was performed at Burleson Research Technologies, Inc. (Morrisville, NC, USA). The data generated were then integrated using two *in silico* ANN DAs to predict LLNA EC3 values.

#### Direct peptide reactivity assay

The covalent binding between a test substance and skin proteins is the molecular initiating event in the AOP for skin sensitization.^9^ The *in chemico* DPRA uses depletion of cysteine and lysine-containing peptides to represent this event. We performed the DPRA test method according to Organisation for Economic Co-operation and Development (OECD) Test Guideline 442C^10^ with few modifications. All IT compounds were tested at 100 mM in acetonitrile (DCOIT, BBIT, MIT, and OIT), water (CMIT/MIT), or acetonitrile:water 1:1 v/v (BIT).

Concentrations were adjusted for measured purity. CMIT/MIT was a mixture of CMIT (10.8%) and MIT (3.4%), thus a combined purity of 14.2% was used with a weighted molecular weight (MW) of 141.36 [(0.761 × 149.592) CMIT + (0.239 × 115.15) MIT] in accordance with Test Guideline 442C. A positive control of 100 mM cinnamic aldehyde (CASRN 104-55-2) in acetonitrile was used to assess run acceptance. The purity of the synthetic peptides used in the assay, acetylated lysine (Ac-RFAKAA-COOH), or acetylated cysteine (Ac-RFAACAA-COOH) (RS Synthesis, Louisville, KY, USA) was 98% or higher.

IT working solutions were combined with the cysteine-containing peptide at a ratio of 1:10 and with the lysine-containing peptide at a ratio of 1:50. These solutions were incubated for 24 hours in the dark at 25°C ± 2.5°C. The concentrations of the peptides were then measured using high-performance liquid chromatography with gradient elution and UV detection at 220 nm (Shimadzu LC-2010AHT). Data analysis was performed with LabSolutions version 5.97. The percent depletion of the lysine or cysteine peptide was calculated relative to peptides incubated with solvent and then the two depletions were averaged (Avg.Lys.Cys). To assess potential quantitative interference of the test compound with UV monitoring, we also assayed the test substance without cysteine or lysine peptide stock. We classified a test substance as positive if Avg.Lys.Cys was >6.38% and assigned relative reactivity categories per Test Guideline 442C.

#### KeratinoSens

KeratinoSens is an in vitro skin sensitization assay that addresses key event 2 of the AOP, which is relevant to keratinocyte responses, including activation of inflammatory cytokines and induction of cytoprotective genes. The KeratinoSens human keratinocyte cell line was kindly provided by Dr. Andreas Natsch (Givaudan Schweiz AG, Switzerland). The KeratinoSens assay was performed as described by Settivari et al^11^ and adopted by OECD in Test Guideline 442D^12^ with minor modifications. To make stock solutions, all compounds were dissolved in dimethyl sulfoxide (DMSO) at 200 mM (BIT, CMIT/MIT, and MIT), 50 mM (OIT and BBIT), or 6.25 mM (DCOIT) based on the results of solubility testing. Starting concentrations were 100-fold dilutions of the stocks, producing concentrations of 2000, 500, and 62.5 μM, respectively.

The CMIT/MIT mixture was prepared according to Test Guideline 442D instructions for testing chemicals without a defined MW by preparing a default concentration of 40 mg/mL and assuming an MW of 200 g/mol and purity of 100% to prepare the “200 mM” working solution. Stock solutions were adjusted for purity with the CMIT/MIT using a combined purity of 14.2% and weighted MW of 141.36 [(0.761 × 149.592) CMIT + (0.239 × 115.15) MIT]. Since CMIT/MIT was adjusted by the combined MW of its constituents, the starting concentration was calculated to 401.8 mM. This was done by multiplying the concentration (2000 μM) by the combined purity (0.142) and dividing the product by a correction factor of the weighted MW (0.7068) representing the assumed MW ratio (141.36 ÷ 200 g/mol). Each solution was tested at 12 concentrations prepared as twofold serial dilutions of the starting concentration.

The positive control was a twofold dilution of cinnamic aldehyde in DMSO at five concentrations ranging from 4 to 64 μM. The negative control was complete Dulbecco's modified Eagle's medium supplemented with 9.1% fetal bovine serum and 0.55 mg/mL Geneticin^®^ (GIBCO) with 1% DMSO. A no-cell blank was also included. Luminescence was measured with a SpectraMax^®^ i3 spectrophotometer (Molecular Devices) and analyzed by SoftMax^®^ Pro GxP version 6.5.1 (Molecular Devices) to assess luciferase activity.

The average maximum fold induction of luciferase activity observed at any test substance concentration and the positive control were determined, as well as the test material concentration that increased luciferase activity 1.5-fold (EC_1.5_). In addition, cell viability was determined in a separate plate using the MTT assay, in which reduction of the yellow tetrazolium dye [3-(4,5-dimethylthiazol-2-yl)-2,5-diphenyltetrazolium bromide] to a purple formazan product was assessed by measuring absorbance with a spectrophotometer (SpectraMax i3 and SoftMax Pro GxP version 6.5.1).

IT compound dilutions were tested as a single well in two or more independent 96-well plates (represents intra-assay replication) run concurrently as part of a single assay. This schema was repeated at least two times (represents inter-assay replication) for a prediction to be made. Each assay was considered acceptable when all of the following criteria were met: (1) the positive control induced a dose-dependent increase in luciferase activity with EC_1.5_ between 7 and 30 μM; (2) maximum luciferase induction of the positive control at 64 μM was between twofold and eightfold; and (3) average coefficient of variation of the luminescence reading for the solvent-control wells was <20%. In accordance with OECD Test Guideline 442D,^12^ a test substance was considered positive for skin sensitization when the following conditions were met:

- Average maximum fold induction of luciferase activity was at least 1.5-fold over the solvent-control value.
- Cell viability was >70% at the lowest concentration that induced an increase in luciferase activity of at least 1.5-fold over control.
- The EC_1.5_ value was >1000 μM.
- There was a dose-dependent increase in luciferase induction.

#### Human cell line activation test

The h-CLAT is an in vitro assay that addresses key event 3 of the AOP for skin sensitization, the activation and mobilization of dendritic cells including induction of inflammatory cytokines and surface molecules leading to T cell priming. The h-CLAT test method was performed according to OECD Test Guideline 442E.^13^ The human monocytic leukemia cell line THP-1 (American Type Culture Collection, Manassas, VA, USA) was used to conduct the assay. Flow cytometry was used to measure expression of CD86 (alternate name B7.2; clone FUN-1) and CD54 (intercellular adhesion molecule 1) cell surface markers associated with dendritic cell activation. The positive control was 2,4-dinitrochlorobenzene prepared in DMSO and diluted to 4.0 μg/mL in culture medium.

The negative control was culture medium with the appropriate solvent concentration added. The ITs were prepared based on solubility in either phosphate-buffered saline (PBS) or DMSO at final in-well concentrations up to 5000 μg/mL (0.5%) in PBS or 1000 μg/mL (0.1%) in DMSO. An eight-concentration 1:1.2-fold dose range-finder cytotoxicity assay was conducted using propidium iodide staining to identify the concentration resulting in 75% cell viability (CV75; 25% cytotoxicity). If test substances prepared in PBS were not cytotoxic, the starting concentration was 5000 μg/mL (0.5%). For the main assay, test substances were prepared in either PBS or DMSO at 100-fold (PBS) or 500-fold (DMSO) of 1.2 × CV75 as the starting (highest) concentration. The solvent, starting concentration, and CV75 for each IT compound are provided in Table 2.

**Table 2.** Isothiazolinone Solvent and Starting (Highest) Concentrations Tested in the Human Cell Line Activation Test

Compound	Selected solvent	Starting concentration (μg/mL)	CV75 (μg/mL)
BIT	DMSO	15.7	13.1
CMIT/MIT	PBS	3.65^a^	3.04^a^
OIT	DMSO	10.6	8.8
BBIT	DMSO	4.0	3.3
MIT	PBS	29.5	24.6
DCOIT	DMSO	1.1	0.9

^a^ CMIT/MIT was prepared for the assay assuming a purity of 100%. Using a combined purity of 14.2%, as was utilized for the DPRA, translates the calculated starting concentration of 25.7 to 3.65 μg/mL and calculated CV75 of 21.4 to 3.04 μg/mL.

DMSO, dimethyl sulfoxide; DPRA, direct peptide reactivity assay; PBS, phosphate-buffered saline.

For the main assay, eight 1.2-fold dilutions in the appropriate solvents were made to obtain the stock solutions that were further diluted 50-fold (PBS) or 250-fold (DMSO) into the culture medium as working solutions, then diluted twofold in the plate to reach final in-well concentrations. CD86 and CD54 expression was measured by flow cytometry using fluorochrome-tagged antibodies. The relative fluorescence intensity for each marker, with respect to solvent controls, was determined at each of the eight 1.2-fold dilutions of test material after a 24-hour exposure to the test substance. A BD Accuri™ C6 flow cytometer using CFlow Plus version 1.0.264.21 (BD Biosciences) was used to acquire and analyze viability and cell surface activation marker expression.

Each IT was tested in at least two independent runs to derive a single result based on the CD86 and CD54 expression levels. In accordance with OECD Test Guideline 442E,^13^ a test substance was positive if at least one of the following conditions were met in two independent runs:

- The relative fluorescence intensity for CD86 was ≥150% in at least one tested concentration (with cell viability ≥50%).
- The relative fluorescence intensity for CD54 was ≥200% in at least one tested concentration (with cell viability ≥50%).

If neither condition was met, the test was negative. For substances classified as positive, we determined the effective concentration that induced a relative fluorescence intensity of 150% for CD86 and a relative fluorescence intensity of 200% for CD54. We determined the minimum induction threshold, which was the lower of either of these values.

### DAs used to estimate points of departure

Two Shiseido ANN models were used to integrate data from the *in chemico* and in vitro tests to predict the LLNA EC3.^8^ These are nonlinear statistical models consisting of an input layer (*in chemico* and in vitro results), a hidden layer, and an output layer (EC3 predictions). The DPRA/h-CLAT and DPRA/h-CLAT/ARE models from Hirota et al^8^ were chosen based on availability of the input data and the published performance of the models.^8,14^ The DPRA/h-CLAT model (ANN D_hC) uses quantitative values from DPRA (Avg.Lys.Cys) and h-CLAT (minimum induction threshold). The DPRA/h-CLAT/ARE model from Hirota et al^8^ uses DPRA, h-CLAT, and the maximum response from an in-house antioxidant response element (ARE) test. In this study, we substituted the average maximum fold induction value for luciferase activity from KeratinoSens, which is functionally and mechanistically similar to the ARE for a DPRA/h-CLAT/KeratinoSens model (ANN D_hC_KS).

The ANN DAs were coded in R (version 3.6.2) using *gdata, caret,* and *e1071* packages to process data and analyze results, and the *RSNNS* package to build the neural network models. The inputs and output were log transformed and the ANN models had two hidden layers with five and two nodes, respectively. Logistic activation functions were used for the hidden and output layers and 10,000 iterations were used for training. All weights and bias were initialized with distributed random values, backpropagation with momentum term was used as the learning function, and functions were updated in topological order as is standard for feedforward nets. Learning rate, scaling functions, and momentum parameters were inferred from Hirota et al.^8^ The ANN DAs coded in R were run on the training and test data from Hirota et al^8^ and benchmarked against previously published results produced using the commercial software QwikNet.

The performance of the open-source R versions of the ANN DAs were equivalent to the versions produced using QwikNet, where the ANN D_hC model demonstrated an *R*^2^ of 0.62 and root-mean-squared error of 0.64 (see Fig. 2A from Hirota et al^8^) and the ANN D_hC_KS model had an *R*^2^ of 0.72 and root-mean-squared error of 0.64 (see Fig. 5A from Hirota et al^8^) between predicted and observed EC3 values from the available LLNA data. For each IT compound, each model was run 100 times and mean EC3 and 95% confidence intervals (CIs) were calculated. Additional details on DAs and performance-based validation on a set of 128 reference chemicals can be found in Kleinstreuer et al.^14^ All data used as information sources for the DAs, as well as the DA output predictions, are included in Supplementary File S1. Training/test data and R code to build and evaluate the ANN models are available upon request.

### Evaluation of in vivo reference data

Historical LLNA data obtained from two major sources were used as reference data. One source was a Dow Chemical Company^15^ report submitted to U.S. EPA, which compiled data from 17 studies performed by or for chemical sponsors. The other source was the published literature, which proffered 15 additional studies. Multiple LLNA studies were available for all substances except BBIT, which had no LLNA data. These data were integrated using two approaches, described as follows, to determine a single representative LLNA EC3 value for each substance. These representative EC3 values were used to assign potency classifications using the Globally Harmonized System of Classification and Labelling of Chemicals^16^ (GHS). GHS 1A sensitizers have EC3 ≤ 2%; GHS 1B sensitizers have EC3 > 2% and negative substances are not classified (NC). GHS 1A sensitizers are described as having a high frequency of occurrence or high potency and GHS 1B sensitizers are described has having a low frequency of occurrence or low or moderate potency.

In Approach 1, the LLNA data submitted to U.S. EPA by Dow Chemical Company were evaluated to determine a representative EC3 by selecting a single test for each substance that used acetone or acetone:olive oil 4:1 v/v as the solvent. However, for OIT, two representative tests with similar EC3 values, 0.20% and 0.25%, were selected. These tests were performed by the same laboratory and performed at approximately the same time. Selecting tests with the same or similar solvents may be appropriate for comparing the potencies of the ITs because EC3 values can vary with solvent.^17,18^ Of the studies submitted by Dow Chemical Company, the representative EC3 values using Approach 1 were also the most potent values available for each substance (Table 3). Approach 1 classified all substances with LLNA results as GHS 1A sensitizers.

**Table 3.** Representative Local Lymph Node Assay EC3 Values for Isothiazolinones

Chemical	Approach 1 LLNA EC3 (%)^a^	Approach 1 GHS classification	Approach 2 mean LLNA EC3 (%)^b^	n for Approach 2	Approach 2 GHS classification
DCOIT	0.004	1A	0.008 (0–0.053)	2	1A
CMIT/MIT	0.002	1A	0.018 (0.0011–0.034)	9	1A
OIT	0.2–0.25 (*n* = 2)	1A	0.361 (0.029–0.69)	4	1A
MIT	0.863	1A	1.154 (0–3.476)	3^c^	1A
BIT	1.54	1A	10.57 (0–23.36)	7	1B
BBIT	NA	NA	NA	0	NA

^a^ EC3 values shown in % concentration. *n* = 1 unless otherwise noted.

^b^ EC3 values shown in % concentration. Numbers in parentheses are the 95% CIs for the mean EC3.

^c^ Four acceptable LLNA studies were available for MIT, but one was negative and did not provide an EC3 value.

CI, confidence interval; GHS, Globally Harmonized System of Classification and Labelling of Chemicals; LLNA, local lymph node assay; *n*, number of studies; NA, not available.

Approach 2 used the 17 studies provided by Dow and the 15 studies (32 total) from the literature to determine a representative EC3 for each substance (Supplementary File S2). Again, no studies were found for BBIT, whereas 3–13 studies were available for each of the other five substances. One MIT test with EC3 = 1.9% from Gerberick et al^19^ was excluded because it was also reported by Basketter et al.^20^ It was determined to be duplicated between the two references because it had the same stimulation index values with erroneous test concentrations and EC3 value (explained in Roberts^21^). The remaining LLNA studies were evaluated using the OECD approach for reference data, which accepts tests with 3 or 4 days of chemical application.^22^ To be included, studies must have had these attributes:

- The test substance was applied topically to both ears of the mice.
- Lymphocyte proliferation was measured in the lymph nodes draining the site of test substance application.
- Lymphocyte proliferation was measured during the induction phase of skin sensitization.
- A vehicle control must be included.
- Either individual or pooled animal data were collected.
- Concentrations tested and corresponding stimulation index values are available.
- Administration of ^3^H-methyl thymidine or other radiolabeled marker must be in vivo rather than *ex vivo.*
- Sodium lauryl sulfate was not applied to enhance the response.
- Extrapolated EC3 values passed the criteria from Ryan et al^23^ as follows:
- The lowest measured stimulation index value was <5.
- The extrapolated EC3 was <10-fold of the closest tested concentration.
- The slope ratio was ≤2 and non-negative. This value is the ratio of the slope from the high dose to the mid-dose, to the slope from the mid-dose to the lowest dose.

We thus rejected five studies for Approach 2 because they did not meet the criteria for acceptable extrapolated EC3 values (see Supplementary File S2). This resulted in between two and nine LLNA studies being available for each of the five substances (Table 3). Results of these studies were used to calculate a mean EC3 for each substance, which was used as the representative EC3 for the substance. We also calculated the 95% CI for the mean. If the lower bound of a CI was less than zero, it was censored to zero. Approach 2 classified all substances with LLNA results as GHS 1A sensitizers, except for BIT, which was classified as a GHS 1B sensitizer.

### Data analyses

We determined the concordance of the hazard classifications by comparing classifications derived from the estimated EC3 values from the ANN models with the classifications derived from the representative in vivo LLNA EC3 values from Approaches 1 and 2 described earlier.

## Results

### Skin sensitization hazard comparison of individual nonanimal methods with respect to in vivo results

We used the historical LLNA studies described earlier as reference data for evaluating the nonanimal data; reference LLNA results classified all of the IT compounds as sensitizers except for BBIT, which did not have LLNA data.

For BIT, the DPRA failed to produce a result for lysine peptide depletion because BIT co-eluted with the peptide. However, cysteine depletion values were generated. The DPRA, KeratinoSens, and h-CLAT classified all of the IT compounds as sensitizers. These hazard classifications were concordant with the available LLNA outcomes.

### Skin sensitization potency comparison of individual nonanimal methods with respect to in vivo results

The LLNA EC3 reference values for Approach 1 and Approach 2 and the corresponding GHS classifications used for evaluating the nonanimal results, are shown in Table 3. DCOIT, CMIT/MIT, OIT, and MIT, despite having a wide range of LLNA EC3 values, were all classified as GHS 1A (high frequency of occurrence or a high potency). BIT was classified as GHS 1A (EC3 = 1.54%) using Approach 1 and as GHS 1B (EC3 = 10.57%; low frequency of occurrence or a low or moderate potency) using Approach 2. All five ITs with LLNA data were positive, with EC3 values ranging from 0.004% to 1.54% for Approach 1 and from 0.008% to 10.57% for Approach 2. Approach 1 EC3 values were 1.3- to 9-fold lower than the Approach 2 mean EC3 values and may reflect differences in the acceptability criteria, solvents, and number of studies used in the two data sets. Approach 2, which used multiple EC3 values for each IT to arrive at the mean EC3 as the representative value, provided information on the variability of the EC3 values through the 95% CI.

Although the OECD test guidelines for the nonanimal test methods do not include use of the methods for determining potency of skin sensitizers, we used the measurement endpoints as indicators of potency for comparison with LLNA EC3 values (Table 4). All IT compounds were classified as having high reactivity in the DPRA. The Avg.Lys.Cys depletion values from DPRA were 50%–55.3%. Only cysteine peptide depletion, which was 100%, was obtained for BIT.

**Table 4.** Skin Sensitization Potency for Local Lymph Node Assay and Nonanimal Methods

Chemical	Approach 1 LLNA EC3 (%)	Approach 2 mean LLNA EC3 (%)	DPRA Avg.Lys.Cys (% depletion)^a^	KeratinoSens EC_1.5_ (μM)	h-CLAT minimum induction threshold (μg/mL)^b^
DCOIT	**0.004**	**0.008**	**55.2**	***1.32***	**0.92**
CMIT/MIT	**0.002**	**0.018**	**55.3**	***3.41***	**2.63**
OIT	**0.2–0.25**	**0.361**	**50**	***2.19***	**0.95**
MIT	**0.863**	**1.154**	**50**	***9.54***	*11.6*
BIT	**1.54**	*10.57*	**NA** ^c^	***3.14***	**7.63**
BBIT	NA	NA	**50**	***3.84***	**3.01**

Bold values refers to GHS 1A (or strong) for LLNA and h-CLAT or high reactivity for DPRA; italicized values refer to GHS 1B for LLNA or weak for h-CLAT; bold and italicized values refer to all KeratinoSens tests that met the criteria for a positive response, but potency categorization was not possible.

^a^ Reactivity classes based on OECD Test Guideline 442C.^9^

^b^ Potency categories based on Takenouchi et al.^23^

^c^ Cysteine peptide depletion was 100%, but lysine peptide depletion could not be evaluated because the test substance co-eluted with the lysine peptide peak.

DPRA, direct peptide reactivity assay; h-CLAT, human cell line activation test; NA, not available.

KeratinoSens EC_1.5_ values, which ranged from 1.32 to 9.54 μM, are not associated with potency categories. The h-CLAT minimum induction thresholds ranged from 0.92 to 11.6 μg/mL. According to the sequential testing strategy from Takenouchi et al,^24^ these h-CLAT data categorize all substances except MIT as strong sensitizers because the minimum induction thresholds are 10 μg/mL or less. MIT is classified as a weak sensitizer; however, because its 11.6 μg/mL minimum induction threshold is near Takenouchi et al's cutoff value of 10 μg/mL that distinguishes strong from weak sensitizers.

### Comparison of in vivo and ANN results

The ANN D_hC and ANN D_hC_KS models were used to integrate data from the *in chemico* and in vitro tests, which covered multiple key events of the skin sensitization AOP, to predict the LLNA EC3. The ANN D_hC model used DPRA and h-CLAT, and the ANN D_hC_KS model used DPRA, h-CLAT, and KeratinoSens. Both models predicted that all six ITs would be sensitizers, which was concordant with the in vivo LLNA results. Specific to potency, both ANN DAs classified the ITs as 1A sensitizers. In comparison, the in vivo GHS potency classification was 1A for each of the ITs based on Approach 1 or Approach 2 LLNA EC3 values, except for BIT, which was either 1A (Approach 1) or 1B (Approach 2) (Table 4).

**FIG. 1.**
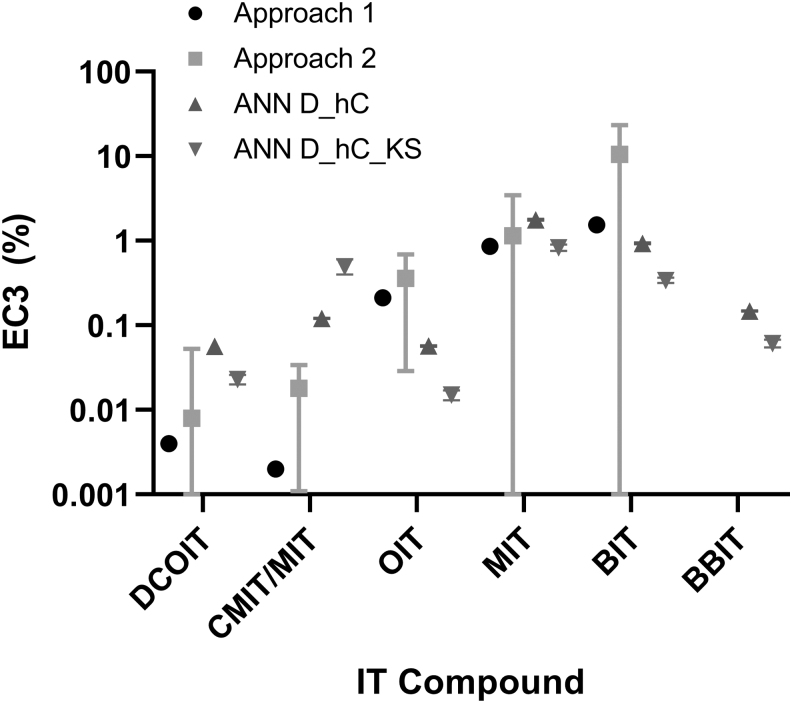
EC3 values for ITs from LLNA and predicted by ANN DAs. Four values are shown for each chemical except BBIT, which had two values. Approach 1 and Approach 2 are in vivo LLNA values; ANN D_hC and ANN D_hC_KS are *in silico* predictions from the ANN DAs. All EC3 values represent percent concentration. Error bars show 95% CIs. Lower confidence limits that were less than zero were censored to zero. Approach 1 has no CIs because only one study was selected for the representative value (except for OIT, for which two studies were selected). ANN, artificial neural network; CIs, confidence intervals; DAs, defined approaches; LLNA, local lymph node assay.

The root mean square error across Approach 1 LLNA EC3 values and the predicted *in silico* EC3 values was 0.49 for the ANN D_hC model, which increased to 0.57 for the ANN D_hC_KS model. The mean absolute error was 0.36 for ANN_D_hC and 0.38 for ANN D_hC_KS model. Across Approach 2 LLNA EC3 values and the ANN DA EC3 values, the root mean square error was 4.32 for the ANN D_hC model and 4.58 for the ANN D_hC_KS model, and the mean absolute errors were 2.14 and 2.28, respectively. Differences among these values were driven by magnitude of the LLNA EC3 values between Approach 1 and Approach 2 results for BIT. Although the in vivo and *in silico* CI for BIT did overlap (Fig. 1), the average EC3 predictions derived from the DAs were closer to the LLNA value from Approach 1 than that from Approach 2.

The *in silico* EC3 predictions from the two ANN DAs are shown with the LLNA EC3 values in Table 5 and Figure 1. The mean predicted EC3 values from the ANN D_hC model and the ANN D_hC_KS model varied from one another by 2.1- to 4.1-fold. The largest discrepancy (4.1-fold) was between the EC3 predictions of the two ANN DAs for the CMIT/MIT mixture. With the exception of those for CMIT/MIT, the EC3 predictions from the ANN D_hC_KS model were lower (i.e., indicating higher potency) than those from the ANN D_hC model. The mean EC3 predictions from the two ANNs had narrow 95% CIs that did not overlap with one another (Fig. 1). The upper bounds of the intervals varied from the lower bounds by only 1.2-fold to 1.5-fold. The ANN DA EC3 predictions had overlapping 95% CI with the Approach 2 LLNA EC3 values, with the exception of CMIT/MIT (Fig. 1), which had a 3.5-fold lower bound of the CI for the ANN D_hC DA. Approach 1 LLNA EC3 values had no CIs because they were single values.

**Table 5.** Comparison of *In Silico* Artificial Neural Network EC3 Predictions with In Vivo EC3s for Isothiazolinones

Chemical	Approach 1 LLNA EC3 (%)	Approach 2 mean LLNA EC3 (%)	ANN D_hC*^a^* predicted EC3 (%)	ANN D_hC_KS*^b^* predicted EC3 (%)
DCOIT	0.004	0.008 (0–0.053)	0.0566 (0.0555–0.0578)	0.023 (0.02–0.026)
CMIT/MIT	0.002	0.018 (0.0011–0.034)	0.121 (0.119–0.123)	0.492 (0.4–0.605)
OIT	0.2–0.25	0.361 (0.029–0.69)	0.0569 (0.0559–0.058)	0.015 (0.013–0.017)
MIT	0.863	1.154 (0–3.476)	1.775 (1.732–1.818)	0.826 (0.759–0.9)
BIT	1.54	10.57 (0–23.36)	0.934 (0.909–0.959)	0.341 (0.317–0.367)
BBIT	NA	NA	0.148 (0.146–0.151)	0.061 (0.055–0.068)

All EC3 values represent percent concentration. Ranges in parentheses are 95% CIs. Lower confidence limits that were less than zero were censored to zero. Approach 1 has no CIs because only one study was selected for the representative value (except for OIT, for which two studies were selected).

^a^ Model 1 from Hirota et al,^8^ which uses h-CLAT and DPRA.

^b^ Model 4 from Hirota et al,^8^ which uses h-CLAT, DPRA, and KeratinoSens.

ANN, artificial neural network; NA, not available.

## Discussion

We tested six IT compounds that are suspected or known sensitizers as a case study to evaluate nonanimal DAs for skin sensitization that can provide point-of-departure estimates for use in quantitative risk assessment. We quantitatively assessed skin sensitization without using animals by applying two *in silico* approaches (i.e., ANN models developed by Hirota et al^8^) to data generated by *in chemico* and in vitro skin sensitization assays.

As stand-alone methods, the DPRA, KeratinoSens, and h-CLAT provided hazard classifications that were consistent with the LLNA classification of these substances as sensitizers. The results of the DPRA and h-CLAT were also consistent with the strong sensitization potency of these substances indicated by in vivo data. The *in silico* models estimate LLNA EC3 values, which can be used for hazard and potency classification and as points of departure for risk assessment. In this study, we found the *in silico* EC3 predictions to be much less variable than LLNA EC3 values, which were available for five of the IT compounds. Four of the five IT compounds with LLNA EC3 values had in vivo CIs that overlapped the CIs derived from the *in silico* EC3 predictions.

### Considering strengths and uncertainties for DAs and in vivo reference values

Human health risk assessment involves evaluating the strengths and uncertainties of available information as part of a weight-of-evidence evaluation.^25,26^ Here we consider the strengths and uncertainties relevant to the *in chemico*/in vitro/*in silico* ANN and LLNA point of departure estimates.

The strengths of the DPRA, KeratinoSens, and h-CLAT test methods are that they use human-relevant cellular and molecular targets to independently assess the activation of three key events in the AOP for skin sensitization. These *in chemico* and in vitro assays have been internationally validated and harmonized in standardized OECD test guidelines.^10,12,13^ Although not validated as stand-alone tests for skin sensitization hazard, they are acceptable for regulatory use when used with other information, such as the ANN models employed herein. Reproducibility for these test methods is at least 80%, which is higher than in vivo results.^10,12,13^ However, there are qualitative uncertainties for the *in chemico* and in vitro test methods, including the fact that they only assess the first three key events of the AOP. They do not assess the fourth key event, T cell proliferation, or the adverse outcome, allergic contact dermatitis. They also do not consider test substance pharmacokinetics (i.e., absorption, distribution, metabolism, or excretion).

The ANN models used in this effort have been published by OECD as a case study on the use and documentation of DAs for regulatory purposes.^27^ The ANNs incorporate information from each of these human-relevant *in chemico* and in vitro tests to account for the first three key events of the AOP for skin sensitization without using animals. Test method performance for these approaches is as good or better than the LLNA. A study using data for 126 chemicals^14^ showed that human potency classifications were predicted by the LLNA with an accuracy of 59% with 20% overpredicted and 21% underpredicted. In comparison, the ANN D_hC had an accuracy of 61% with 22% overpredicted and 17% underpredicted, and the ANN D_hC_KS had an accuracy of 63% with 25% overpredicted and 12% underpredicted. However, an element of qualitative uncertainty for both ANN models is that they were trained to predict T cell proliferation results in mice (EC3 values)^8^ and not the adverse outcome in humans, the species of interest.

Our confidence in ANN D_hC_KS is higher than that for ANN D_hC because ANN D_hC_KS involves three key events of the skin sensitization AOP, whereas ANN D_hC involves only two key events. Specifically, the ANN D_hC DA incorporates DPRA and h-CLAT data, whereas the ANN D_hC_KS incorporates KeratinoSens. The mean EC3 values reported for the ANN DAs result from 100 runs in each model. A quantitative measure of uncertainty is provided by the 95% CIs around the mean ANN EC3 values that results from variation inherent in the machine learning algorithm.

An important strength of the LLNA is that it incorporates all the biological processes of absorption, distribution, elimination, and toxicodynamics that are relevant to test substance exposure. However, although the LLNA is an internationally harmonized test method validated to assess skin sensitization hazard and potency,^28^ there are uncertainties associated with the LLNA EC3 values. Qualitative uncertainties regarding the LLNA EC3 data are similar to those of the ANN models. Specifically, although the LLNA incorporates all four key events of the AOP, it does not directly measure the adverse outcome of skin sensitization. In addition, mice, the experimental model used in the LLNA, are not humans, the species of interest.

The reproducibility of the LLNA presents an opportunity for improvement when considering nonanimal approaches. The inherent reproducibility of the LLNA is 70%–80% for hazard classification and 60%–70% for potency classification depending on the summary statistic (i.e., mean and median) used for comparison.^17,29^ Approach 2 values reported in Table 5 use LLNA EC3 values from tests that meet criteria designed to identify the most reliable EC3 values^22^ and the 95% CIs are included to provide a quantitative measure of the uncertainty in the results. Approach 1 LLNA EC3 values that use the same or similar solvents across the substances and were typically the most potent EC3 values available were based on single studies for each substance (and, therefore, are shown without CIs).

### Using these data for point-of-departure estimates

Dermal contact is a major route of human exposure to IT biocides, and skin sensitization can occur from dermal exposure.^6,30,31^ U.S. EPA-regulated IT biocides are commonly used as materials preservatives in products that do not bear pesticide labels and for which personal protective equipment cannot be required (i.e., treated articles). One member of the IT biocide class widely used as a materials preservative, MIT, has resulted in increasing incidences of contact allergy associated with exposure.^30^

U.S. EPA typically requires that pesticide products containing sensitizers be labeled as such; however, preserved materials such as cleaning products, pressure-treated wood and paint are treated articles, not pesticide products, and they do not have pesticide labeling. Thus, U.S. EPA developed an approach to quantify risk from exposure to sensitizing treated products that could not bear labels.^5^ At that time, the approach used animal-based methods such as the LLNA or results of clinical studies in human volunteers. U.S. EPA used the *in chemico*, in vitro, and *in silico* quantitative approaches reported here to assess skin sensitization risk to IT biocides because, in combination, these nonanimal methods provide information that is more reliable, reproducible, and human-relevant than the historical LLNA data. Therefore, U.S. EPA has used the ANN EC3 DA to derive EC3 values to extrapolate dermal risk for currently registered ITs.^32^

U.S. EPA has proposed using predicted EC3 values from the ANN D_hC_KS model as the points of departure for skin sensitization induction thresholds (Table 5).^32^ For use in this context, predicted EC3 values were transformed to applied doses per skin area using a factor of 250^33^ to convert the % IT applied to μg IT per cm^2^ skin area. In this conversion, the IT percent concentration is multiplied by 10 to convert to μg/μL and by 25 μL (the amount applied to a mouse ear in the LLNA) to convert to μg and then divided by the 1 cm^2^ surface area of a mouse ear to get μg/cm^2^. Applied dose per skin area is considered a more appropriate dose metric for skin sensitization risk assessment.^34,35^

No-expected-sensitization-induction levels (NESILs) for two ITs have been previously published. The Expert Panel for Cosmetic Ingredient Safety, an independent scientific committee that provides technical support for the Personal Care Products Council (U.S.-based), derived a NESIL of 0.83 μg/cm^2^ for CMIT/MIT as a point of departure to assesses the safety of cosmetic ingredients.^36^ The ANN D_hC_KS estimated an EC3 of 0.49% for CMIT/MIT, which is 120 μg/cm^2^ when transformed to the DSA metric. Although the ANN D_hC_KS EC3 estimate for CMIT/MIT was >100 × higher than the NESIL, a higher uncertainty factor would typically be applied to the ANN-derived value during risk assessment because it estimates a laboratory animal effect instead of a human effect.

For MIT, the ANN D_hC_KS estimated an EC3 of 0.83%, which is 210 μg/cm^2^ when transformed to the DSA metric. The Scientific Committee on Consumer Safety, which provides advice on consumer safety to the European Union,^37^ identified a NESIL of 15 μg/cm^2^. Although the ANN D_hC_KS EC3 estimate for MIT was 14 × higher than the NESIL, higher uncertainty factors would typically be applied to the ANN-derived value during risk assessment because it estimates a laboratory animal effect instead of a human effect.

## Conclusion

The availability of the quantitative *in chemico*, in vitro, or *in silico* approaches reported here provide the opportunity to assess the risk of skin sensitization induction by exposure to IT biocides without using animals. U.S. EPA determined that the in vitro and *in chemico* studies provide information that is more reliable, reproducible, and human-relevant than the historical LLNA data. Our results show that these DAs can be used to support hazard and potency classification and quantitative risk assessment. However, as the adverse outcome is allergic contact dermatitis in humans, development of statistical models to estimate the dose that sensitizes a specific portion of the human population is also underway.^38^

## Supplementary Material

Supplemental data

Supplemental data
